# An unusual case of dysuria, pollakisuria, and eosinophilia: Answers

**DOI:** 10.1007/s00467-021-05130-8

**Published:** 2021-11-18

**Authors:** Fabian Eibensteiner, Ursula Tonnhofer, Alexander Springer, Hubert Kogler, Leila Ronceray, Azadeh Hojreh, Christoph Aufricht, Krisztina Rusai

**Affiliations:** 1 Division of Pediatric Nephrology and Gastroenterology, Comprehensive Center for Pediatrics, Medical University of Vienna, Waehringer Guertel 18-20, 1090 Vienna, Austria; 2 Division of Pediatric Surgery, Comprehensive Center for Pediatrics, Medical University of Vienna, Vienna, Austria; 3 Department of Pediatrics and Adolescent Medicine, St. Anna Children’s Hospital, Medical University of Vienna, Vienna, Austria; 4 Department of Biomedical Imaging and Image-guided Therapy, Medical University of Vienna, Vienna, Austria

**Keywords:** Eosinophilic cystitis, Celiac disease, Eosinophilia, Cystitis, Cystic mass, Pediatric

## Answers

1. **What is the diagnosis and what is its most likely etiology?**

The patient has eosinophilic cystitis, which developed due to eosinophilia triggered by celiac disease.

This is the first case in the current literature of a pediatric patient with celiac disease and concomitant symptomatic eosinophilic cystitis. Until now, only associations of celiac disease with peripheral blood eosinophilia and eosinophilic esophagitis in pediatric patients, as well as one association of an adult patient with eosinophilic cystitis, have been reported [1, 2].

The true etiology in many cases of eosinophilic cystitis remains elusive to this day, with several associations with hypereosinophilic syndromes, allergies, medications, and even with bladder trauma, especially in response to bladder catheterization [3, 4]. Under normal conditions, eosinophils are mainly found in the bone marrow, lymphoid organs, the mucosa of the gastrointestinal tract, and the uterus, but very rarely in other organ tissues. Secondary (reactive) eosinophilia is mainly driven by cytokines, whereas primary (clonal) forms of eosinophilia are mostly caused by tyrosine kinase gene fusions, involving the coding genes for platelet-derived growth factor receptor alpha (*PDGFRA*), beta (*PDGFRB*), or fibroblast growth factor receptor 1 (*FGFR1*). Secondary (reactive) eosinophilia has multiple causes, such as allergy, drugs, and parasitic disease, and respiratory, gastrointestinal, and rheumatologic disorders [1, 5, 6].

Overall, eosinophilic cystitis is a rare inflammatory disorder, especially in the pediatric population [3, 4]. It should be noted that the age of disease onset in the case presented here is very early (at 3 years of age) in contrast to currently published case reports, with a median age of disease onset of 6.5 years [3, 6–8].

2. **What further examinations should be performed?**

Irrespective of its cause, prolonged or marked activation of eosinophils may lead to migration of eosinophils into other organ tissues, such as the heart, lung, skin, or urinary tract, resulting in tissue and subsequent end-organ damage. Therefore, evaluation for the presence of other end-organ damage is essential. Based on individual signs and symptoms, chest X-ray, electrocardiogram, echocardiography, abdominal ultrasound, tissue biopsies, and other evaluations should be performed [1, 5, 6].

We have investigated the peripheral blood for genetic mutations (*PDGFRA*, *PDGFRB*, *FGFR1*) for the most frequent primary (clonal) eosinophilic disease, which yielded negative results [1]. Thorough workup for other secondary end-organ damages did not reveal further involvement of other organs.

In the absence of an underlying cause of eosinophilia, a bone marrow punction should be performed to rule out a hematological neoplasm.

3. **What is the treatment for this disease?**

The treatment of eosinophilic cystitis is unfortunately not well established — there have been no controlled studies performed to date.

However, many cases in children are self-limiting [3]. First-line treatments typically involve the removal of the driving reason, followed by the use of antihistamines and corticosteroids. The use of transurethral indwelling catheters and surgical treatment have also been reported, although less frequently than in adults [1].

In the case of our patient, upon diagnosis of celiac disease, treatment with a gluten-free diet was initiated. This led to a significant decrease and then to normalization of peripheral eosinophila (3.7% of peripheral leukocytes and 0.3 × 10^9^/L absolute eosinophil count) over the course of 3 months, and a decrease of bladder wall thickening to 0.6 cm after 6 months on a gluten-free diet. After 9 months of the gluten-free diet, initial symptoms for eosinophilic cystitis were completely relieved, and urinary bladder wall thickening remained at 0.6 cm.

## Discussion

In the case presented here, eosinophilia may be caused by previously undetected celiac disease, leading to secondary (reactive) eosinophilia and subsequent symptomatic end-organ damage of the urinary bladder wall before distinct symptomatic presentation of the underlying gastrointestinal disorder. The percentage of peripheral eosinophilia in patients with celiac disease was reported in small case series only, with 4/10 and 3/7 patients with celiac disease, having peripheral eosinophilia, and eosinophilic esophagitis [9, 10]. An initial secondary (reactive) eosinophilia of 28% of peripheral leukocytes probably led to urinary bladder wall infiltration with inflammation causing bladder wall thickening up to 2 cm, which clinically correlated well with the boy’s symptoms at the time of diagnosis. This hypothesis is supported by only intermittent recovery upon symptomatic treatment with oxybutynin, and prolonged remission of symptoms, bladder wall formations, and peripheral blood eosinophilia after initiation of a gluten-free diet.

Prognosis of eosinophilic cystitis is difficult to establish, due to very few pediatric cases worldwide. However, most cases are responsive to various treatments, while patients with prior allergic conditions and granulomatous diseases show high rates of recurrence [3]. Therefore, we currently keep this patient on close follow-up at 3-month intervals at our outpatient clinic.

## Conclusions

In conclusion, we are the first to report the case of a 3-year-old boy with celiac disease causing symptomatic eosinophilic cystitis before the onset of gastrointestinal symptoms. Treatment of the underlying condition (celiacfig disease) has led to the resolution of eosinophilia and eosinophilic cystitis.

## Data Availability

No data or additional material is made available.

## Abbreviations

PDGFRA: Platelet-derived growth factor receptor alpha
PDGFRB: Platelet-derived growth factor receptor beta
FGFR1: Fibroblast growth factor receptor 1

## Code availability

No custom codes were used for this study.
